# Case of Third Dentition in Early Life

**Published:** 1848-07

**Authors:** A. Berry

**Affiliations:** Cincinnati


### CASE OF THIRD DENTITION IN EARLY LIFE.

BY A. BERRY, D. D. S.—CINCINNATI.

In the summer of 1845, I met with a remarkable case of third dentition, of which, having received a minute account, I made the following hasty note in my diary:

"Miss M. E. H., of Hind's Co., Miss., lost all her inferior incisores from ptyalism at two and a half years of age. They were succeeded in a few weeks by the permanent incisores.—

These she had the misfortune to break out by a fall on an andiron when she was four and a half years old. In six or eight months from that time four little bulbs, resembling cartilage, were discovered with apparently slight attachments to the gums where the teeth had been lost. The laterals soon came off and were found to be mere shells. The centrals continued to increase for three or four years, when they had become of the usual size.

The young lady is now twelve years of age. The teeth of third dentition are as firm in their sockets as teeth standing so far apart usually are; evidently of soft texture, as are all her teeth, and one of them with caries on its anterior surface."

Being acquainted with the parents of the young lady, I have no doubt of the correctness of their report. That the vis medicatrix naturæ should sometimes replace other teeth for the permanents, when lost by young persons, is not more unaccountable than that it should do it for the aged.

